# Low-pressure self-expandable metal stent insertion for obstructive colon cancer using water and gel immersion

**DOI:** 10.1055/a-2433-0576

**Published:** 2024-10-16

**Authors:** Takahiro Muramatsu, Masakatsu Fukuzawa, Midori Mizumachi, Satoshi Shimai, Miki Wada, Kazuki Yamamoto, Takao Itoi

**Affiliations:** 1 38548 Department of Gastroenterology and Hepatology, Tokyo Medical University Hospital, Tokyo, Japan

Self-expandable metal stents (SEMSs) are commonly used as a bridge-to-surgery or palliative treatment for obstructive colorectal cancer 1 . Although technical and clinical success rates are high, adverse events such as perforation, migration, and sepsis 2 3 4 may occur owing to the poor visual field due to stool and failure to identify the luminal opening of the tumor, air over-insufflation, and unreasonable guidewire manipulation. Gel immersion can be used to improve the endoscopic view 5 . Herein, we describe a SEMS insertion with a clear view and lower intraluminal pressure using water and gel immersion ( Video 1 ).

Low-pressure insertion of a self-expandable metal stent for obstructive colon cancer using water and gel immersion.Video 1

A 55-year-old woman presented with abdominal pain and nausea. She was diagnosed with bowel obstruction to sigmoid colon cancer ( Fig. 1 **a** ), and a SEMS was inserted as a bridge-to-surgery treatment. First, we removed the gas from the lumen and filled it with water to create underwater conditions ( Fig. 2 **a, b** ). Because the visual field was poor due to stool and residue, gel was injected (VISCOCLEAR; Otsuka Pharmaceutical Factory, Inc., Tokushima, Japan). The visual field was cleared, and the endoscope reached the tumor ( Fig. 2 **c, d** ). As the tumor was covered with stool and mucus, it was gently washed with water and gel, and the luminal opening was identified ( Fig. 1 **b** , Fig. 2 **e–g** ). Subsequently, the catheter was inserted into the stricture and the proximal colon was confirmed via contrast ( Fig. 2 **h** ). A wire-guided biopsy was then performed; however, bleeding occurred. The gel injection reduced the momentum of bleeding and improved the endoscopic view ( Fig. 2 **i–l** ). Finally, the stent was successfully inserted (22 × 120-mm Niti-S Enteral Colonic Uncovered Stent; Taewoong Medical Co., Ltd., Seoul, Korea) ( Fig. 2 **m–o** ).

**Fig. 1:**
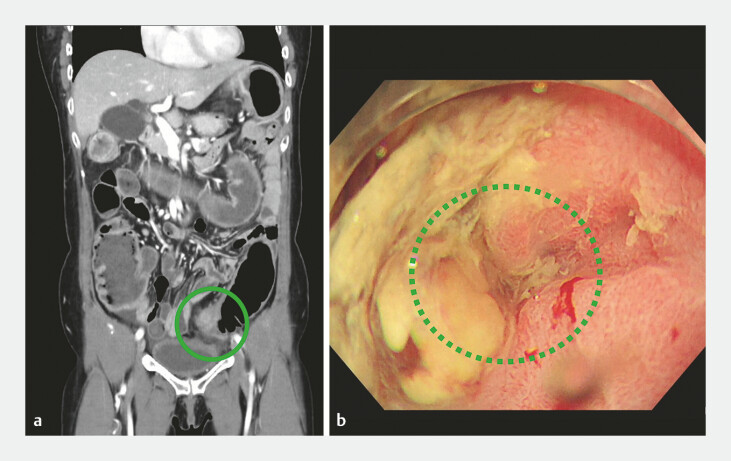
Computed tomography (CT) and endoscopic image of sigmoid colon cancer. **a** The CT image shows wall thickening of the sigmoid colon (green circle) and dilation of the proximal colon. **b** The luminal opening of the tumor (green dotted circle).

**Fig. 2:**
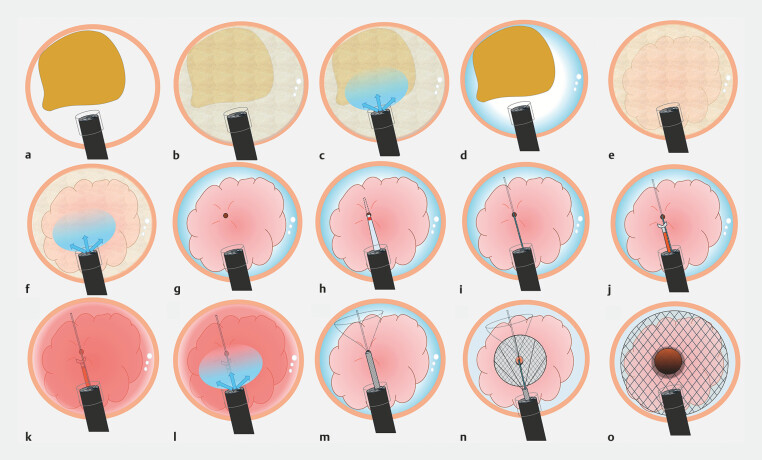
Schema of the low-pressure insertion of a self-expandable metal stent using water and gel immersion. **a** View under gas. **b** Removal of the gas from the lumen and filling it with water. **c** Injecting the gel. **d** The endoscopic view clearly changes. **e** The tumor is covered with stool and mucus. **f** The tumor is gently washed with water and gel. **g** The luminal opening is identified. **h** The catheter is inserted into the stricture. **i** A guidewire is placed. **j** Biopsy of the tumor. **k** Bleeding occurs and negatively impacts the endoscopic view. **l** Injecting the gel. **m** A colonic stent is deployed. **n** Careful deployment of the stent continued. **o** The stent is inserted successfully.

In conclusion, low-pressure insertion of a SEMS with water and gel immersion may prevent air over-insufflation and ensure a good endoscopic field view. This method may reduce patient discomfort and enable safe stent insertion.

Endoscopy_UCTN_Code_TTT_1AQ_2AF

## Notes

Endoscopy E-Videos https://eref.thieme.de/e-videos
*E-Videos* is an open access online section of the journal *Endoscopy* , reporting on interesting cases and new techniques in gastroenterological endoscopy. All papers include a high-quality video and are published with a Creative Commons CC-BY license. Endoscopy E-Videos qualify for HINARI discounts and waivers and eligibility is automatically checked during the submission process. We grant 100% waivers to articles whose corresponding authors are based in Group A countries and 50% waivers to those who are based in Group B countries as classified by Research4Life (see: https://www.research4life.org/access/eligibility/ ). This section has its own submission website at https://mc.manuscriptcentral.com/e-videos .
